# Comparison of Methoxypyrazine Content and Expression Pattern of *O*-Methyltransferase Genes in Grape Berries and Wines from Six Cultivars (*Vitis vinifera* L.) in the Eastern Foothill of the Helan Mountain

**DOI:** 10.3390/plants11121613

**Published:** 2022-06-20

**Authors:** Yanxia Zhang, Xiangyi Li, Xufeng Guo, Ning Wang, Kangqi Geng, Dongmei Li, Zhenping Wang

**Affiliations:** 1 Department of Plant Biology, School of Life Sciences, Ningxia University, Yinchuan 750021, China; zhangyanxiaedu@163.com (Y.Z.); gengkangqi@163.com (K.G.); 2 Department of Food and Wine, College of Food Science and Nutritional Engineering, China Agricultural University, Beijing 100083, China; lixiangyi@sjtu.edu.cn; 3 Department of Horticulture, School of Agriculture, Ningxia University, Yinchuan 750021, China; jerry19941117@icloud.com (X.G.); wnlwyn2020@163.com (N.W.)

**Keywords:** wine grapes, berry quality, methoxypyrazine, *VvOMTs*

## Abstract

Methoxypyrazines (MPs) are a unique class of volatile compounds containing nitrogenous heterocyclics that impart green bell, vegetal and herbal odors to red grape berries and wines. In this study, the quality and MPs levels of grape berries from six representative red wine grape varieties were determined in the two consecutive years. The results showed that, at maturity, the highest total soluble solid was observed in Petit Verdot grape berries in the two consecutive years. While the anthocyanin content showed the highest in Marselan berries in 2018, in 2019, Petit Verdot berries had the highest anthocyanin content. Moreover, 2-methoxypyrazine (MOMP), 3-methyl-2-methoxypyrazine (MEMP) and 3-ethyl-2-methoxypyrazine (ETMP) levels were relatively lower, with almost no detectable in berries at maturity. The relative higher 3-isobutyl-2-methoxypyrazine (IBMP) content was observed in Cabernet Sauvignon, Marselan, Merlot, and Malbec berries. However, 3-sec-butyl-2-methoxypyrazine (SBMP) and IBMP were only detected in six wines, and their levels were higher than those in the grape berries. Furthermore, correlation analysis showed that there was a statistically positive correlation between the expression levels of *VvOMT1* and *VvOMT3* and MPs content in grape berries, while the lowest association was found in the *VvOMT2*. These findings provide a basis for selecting the most suitable grape varieties to improve wine quality.

## 1. Introduction

Methoxypyrazines (MPs) are nitrogen-containing heterocyclic volatile compounds that are associated with the distinct herbaceous/bell pepper characteristics of red wines. MPs can be detected in various plant species, such as peas, asparagus, tomatoes, potatoes, and lettuces [1]. With an earthy flavor, they are the most important representative aromatic compounds in some vegetables and grape varieties [2,3]. Buttery found that 3-isobutyl-2-methoxypyrazine (IBMP) have a fresh green bell pepper aroma, and it has the lowest odor thresholds in water solution [4]. Several studies were conducted on Sauvignon Blanc, Merlot grapes, Vidal, and Cabernet Sauvignon wine, and the results showed that MPs had an effect on wine aroma and flavor, and Sauvignon Blanc white grape varieties had a unique aroma, which may be associated with MPs levels [5,6,7]. However, it is difficult for consumers to accept the high herbaceous odor, which is unfavorable to the development of the wine industry [8,9].

At present, MPs have commonly been identified in a wide range of plants, foods, and microbes. Six kinds of MPs, including 2-methoxypyrazine (MOMP), 3-methyl-2-methoxypyrazine (MEMP), 3-ethyl-2-methoxypyrazine (ETMP), 3-isobutyl-2-methoxypyrazine (IBMP), 3-isopropyl-2-methoxypyrazine (IPMP), and 3-sec-butyl-2-methoxypyrazine (SBMP), have been found in the different species [10]. The concentration of MPs in grape berries and wine are generally lower (2–30 ng/L) [11,12]. The sensory thresholds of these compounds are about 1–2 ng/L in water and 2–16 ng/L in wines [13]. For some white wines, such as Sauvignon Blanc, Riesling, Chardonnay, and Semillon, MPs, as a plant characteristic odor, are acceptable to consumers [11,14,15]. However, MPs can lead to undesirable sensory defects for red wines, such as Cabernet Sauvignon, Merlot, Cabernet Franc, and Pinot Noir [15,16,17]. Normally, MPs levels in wines are closely related to that of grape berries. Therefore, it is important to understand the accumulation and degradation pattern of MPs in grape berries and the content of wines.

Different MPs composition and levels were observed in different grapevine cultivars and may lead to the different levels of odor [17,18]. MPs or their precursors content in grape berries depend on their genotypes. It was reported that 29 grapevine cultivars and berries from Davis, CA were analyzed, and IBMP was only detected in Cabernet Franc, Cabernet Sauvignon, Merlot, Sauvignon Blanc and Semillon [11]. No IBMP was detected in the berries of Muscat Blanc clusters grafted onto Cabernet Sauvignon vines. However, IBMP was detected in all Cabernet Sauvignon berries, regardless of grafting structure at maturity. These data suggested that IBMP or its precursors originate in berries and that its formation depends on the genotype of the grape [11]. More than 50 red wines such as Cabernet Sauvignon, Cabernet Franc, and Merlot have obvious green odor [19].

At present, there are two hypothetical pathways for SBMP, IPMP and IBMP biosynthesis. The first biosynthetic pathway is the amidation of branched amino acids; the biosynthetic precursors of IBMP, IPMP and SBMP could be leucine, valine and isoleucine, respectively. The amidation products were further condensated with α- and β-dicarbonyl compounds, such as glyoxal and acetaldehyde [20]. The second biosynthetic pathway is the MPs synthesis; in the initial step, the condensation between the two amino acids results in a cyclic dipeptide [3]. The two pathways have many similarities, for example, the O-methylation of 2-hydroxy-3-alkyl pyrazine (HP) is the last step in the MPs biosynthesis pathway. However, at present, it is not clear how the intermediate metabolites and MPs are synthesized, and most studies have largely focused on the last step. The activity of *O*-methyltransferase (OMT) in grape berries was closely related to hydroxy pyrazines (HPs) and MPs level. At present, *VvOMT1* and *VvOMT2* genes have been cloned from Cabernet Sauvignon, which are primarily responsible for methylating HPs to MPs [21]. OMT1 could have higher catalytic activity towards 3-Isobutyl-2-hydroxypyrazine (IBHP) than 2-isopropyl-3-hydroxypyrazine (IPHP); however, a completely opposite result was observed in OMT2 [22], while *VvOMT3* had a high affinity for HPs’ precursors [23].

The eastern foothill of the Helan Mountain in Ningxia is considered to be the golden area for grapevine planting in China. This region has unique terroir, such as low precipitation and a large difference in temperature between day and night, which is favorable for the accumulation of secondary metabolites. Therefore, many winemaking grapevine cultivars with high economic value have been gradually introduced to Ningxia. In this study, in order to investigate the quality traits in this region, six common grapevine cultivars, including Merlot, Marselan, Cabernet Sauvignon, Petit Verdot, Pinot Noir, and Malbec, were selected as experimental materials. Firstly, biochemical parameters such as the weight of 100 berries, total soluble solid, titratable acid and anthocyanin content were analyzed in two constitutive years. Moreover, to further understand the changes in MPs in grape berries during their growth and development, MPs content and the expression level of *VvOMTs* were detected in six grapevine cultivars. In brief, the study will provide a theoretical basis for the selection of suitable varieties for Ningxia. This could help provide premium materials for the production of high-quality wines.

## 2. Results

### 2.1. Climatic Conditions

**Figure 1:**
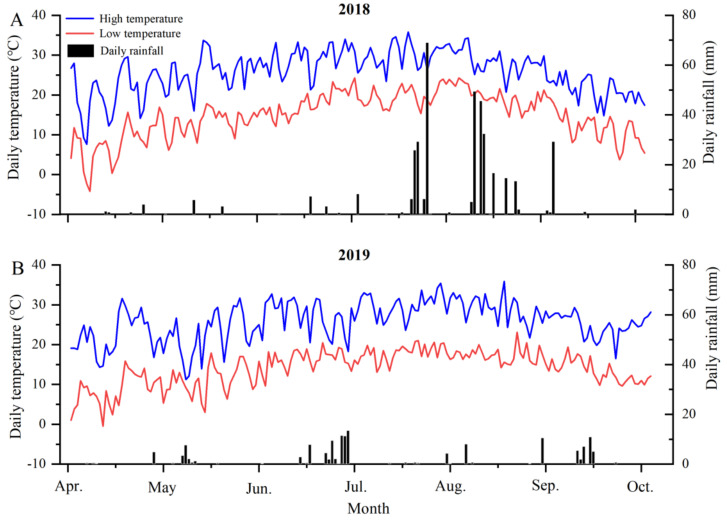
Characteristic of maximum temperature, minimum temperature and daily rainfall from 1 April (budbreak) to 1 October (harvest). (**A**) Climate data of the test site in 2018; (**B**) Climate data of the test site in 2019. The blue lines represent the highest temperature, the red lines represent the lowest temperature, the columns represent the daily rainfall.

The vineyard in the present study was in a winemaking region in the eastern foothill of the Helan Mountain in China. This region has unique ecological characteristics. A meteorological station was installed at Meihe winery. The meteorological data during 2018 and 2019 are presented in Figure 1. The sum of the average daily temperature from 1 April to 31 May was higher for 2018 (1067.7 °C) than 2019 (1015.5 °C). The effective accumulated temperature from April to September was 1906.09 °C in 2018 and 1860.57 °C in 2019, respectively. The rainfall mainly concentrated in summer (July through August) in 2018, and the average annual rainfall was approximately 388 mm, while the average annual rainfall was only 142.8 mm in 2019. Thus, these data indicate that the difference in terroir was significant between the two years (Figure 1A,B).

### 2.2. Analysis of Berry Quality in Different Grape Cultivars

**Figure 2:**
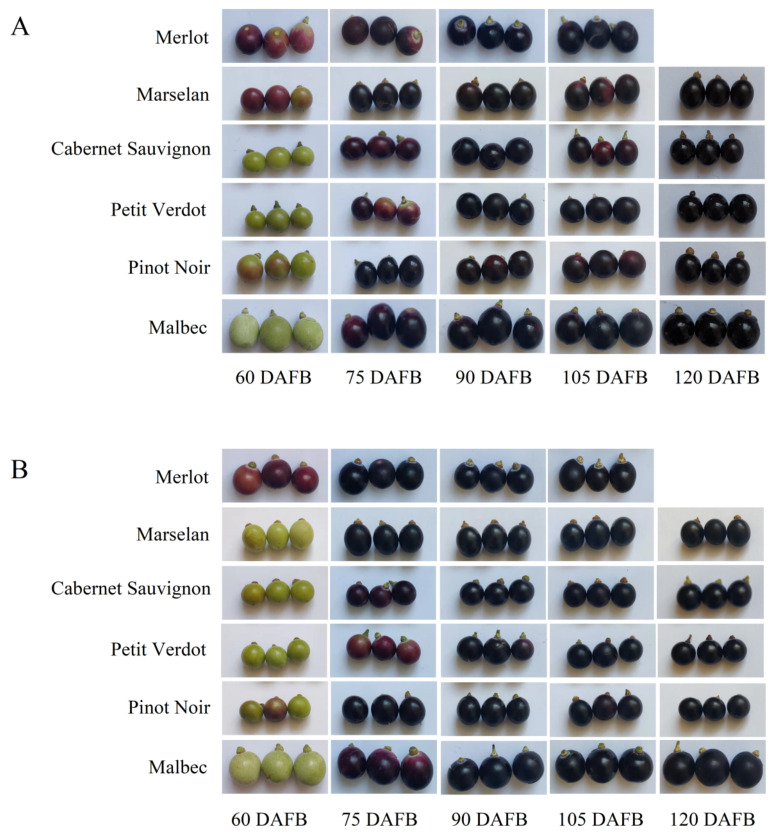
Process of berries development in 6 cultivars. (**A**) Process of Berries development in 6 cultivars in 2018; (**B**) Process of Berries development in 6 cultivars in 2019.

Berry quality is an important determining factor for wine quality. Therefore, firstly, physicochemical indices of grape berries in six cultivars during growth and development were analyzed. The process of grape berries development and ripening is depicted in Figure 2 and used for further analysis. From the visual appearance, we found that the maximum volume could be observed in Malbec berries, while the minimum volume could be observed in Cabernet Sauvignon and Petit Verdot berries. Regrading grape berry development, veraison is defined as the onset of ripening, in which grape clusters begin to color. In this study, at 60 days after full bloom (DAFB), the color of Merlot berries were both changed to red, Pinot Noir was in the early stage of the veraison in 2018 and 2019; however, Cabernet Sauvignon, Petit Verdot and Malbec berries were still green. Meanwhile, by analysis of the appearance of the Marselan berries, we could find that the veraison in 2018 was significantly earlier than that in 2019 (Figure 2A,B).

**Figure 3:**
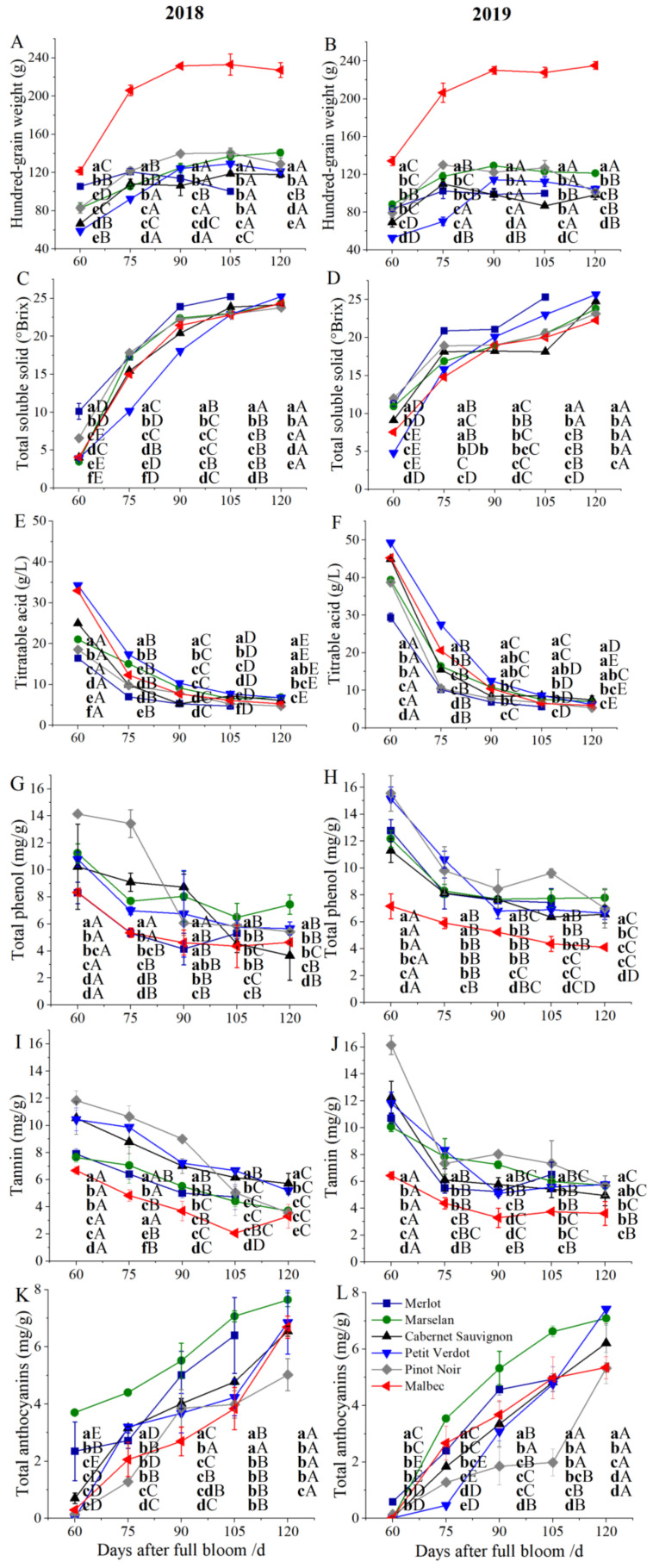
Physicochemical parameters of grape berries from different cultivars. (**A**,**B**) Weight of 100 berries in 2018 and 2019; (**C**,**D**) Total soluble solid (TSS) content of grape berries in 2018 and 2019; (**E**,**F**) Titratable acid (TA) content in 2018 and 2019; (**G**,**H**) Total phenol content in 2018 and 2019; (**I**,**J**) Tannin content in 2018 and 2019; (**K**,**L**) Total anthocyanins content in 2018 and 2019. Three biological replicates were performed. Lines graphs and error bars represent average and SE, respectively. Significant differences were indicated by lowercase letters based on Fisher LSD-test between different grape varieties of the same sampling period. Capital letters were used to indicate significant differences between different sampling periods for the same grape variety (*p* ≤ 0.05).

The weight of 100 berries was measured from 60 to 120 DAFB and the results indicated that it was progressively increased during the development of grape berries, and almost no difference was observed among different cultivars after veraison; however, there was a slight decrease at harvest. Regarding different cultivars, the weight of 100 berries of Malbec was significantly higher than that of other cultivars, while it was at the minimum level in Cabernet Sauvignon and Merlot berries (Figure 3A,B). Moreover, changes in several ripening-related indicators were measured throughout the sampling stages. The total soluble solids (TSS) content of grape berries was progressively increased from 60 to 120 DAFB in all cultivars. The highest TSS content was observed in Merlot berries, up to 3.8% compared with Cabernet Sauvignon berries, followed by Petit Verdot berries, while the lowest TSS content was observed in Pinot Noir berries in 2019, and in Malbec berries in 2018 (Figure 3C,D). A completely opposite trend was observed in the titratable acid (TA). TA content was progressively decreased after 60 DAFB in all cultivars. The highest TA content was found in Marselan berries, but there was no significant difference between Cabernet Sauvignon and Petit Verdot berries. The lowest TA content was observed in Merlot (4.75 g/L) and Pinot Noir (4.75 g/L) in 2018; however, in 2019, TA content of Merlot berries was the lowest. The TA content of Pinot Noir berries was the lowest from veraison to harvest in 2018 and 2019 (Figure 3E,F).

Total phenol content at harvest was the highest in Marselan berries in 2018 and 2019. In two years, the total phenol content of Marselan berries at harvest were 2.04-fold and 1.19-fold compared with the Cabernet Sauvignon berries, respectively, while the total phenol content in Malbec (4.12 mg/g) berries was significantly lower than that in other cultivars in 2019. At harvest, the total phenol content of all cultivars showed no significant differences between 2018 and 2019 (Figure 3G,H). The tannin content of grape berries showed a downward trend with maturity in all cultivars. The highest tannin content was found in Pinot Noir berries, while the lowest tannin content was observed in Malbec berries at 60 DAFB. At harvest, the tannin content in Cabernet Sauvignon berries reached 5.70 mg/g in 2018, which was 1.73-fold than that of Malbec. In 2019, the tannin content of Malbec berries (3.61 mg/g) was significantly lower than that of other cultivars (Figure 3I,J). The anthocyanin content showed an upward trend with the growth and development of grape berries. The highest anthocyanin content was observed in Marselan berries, which was 1.73-fold compared with Cabernet Sauvignon berries at harvest in 2018. In 2019, the content of anthocyanin in Petit Verdot and Marselan berries were 1.19-fold and 1.14-fold higher than that in Cabernet Sauvignon berries, respectively. Anthocyanin content in Pinot Noir berries was the lowest at harvest in consecutive two years (Figure 3K,L). In summary, the overall trend in these quality indices was relatively consistent between 2018 and 2019.

### 2.3. MPs Concentrations in Grape Berries from Different Cultivars

**Figure 4:**
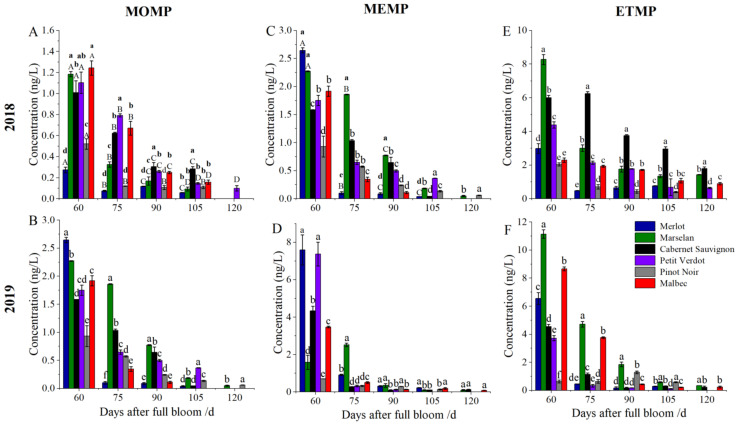
MPs concentrations in grape berries from 6 cultivars during the growth and development. (**A**,**B**) 2-Methoxypyrazine (MOMP) concentration in 2018 and 2019; (**C**,**D**) 3-methyl-2-methoxypyrazine (MEMP) concentration in 2018 and 2019; (**E**,**F**) 3-ethyl-2-methoxypyrazine (ETMP) concentration in 2018 and 2019. Three biological replicates were performed. Three biological replicates were performed. Bars graphs and error bars represent average and SE, respectively. Significant differences were indicated by lowercase letters based on Fisher LSD-test between different grape varieties of the same sampling period. Capital letters were used to indicate significant differences between different sampling periods for the same grape variety (*p* ≤ 0.05).

**Figure 5:**
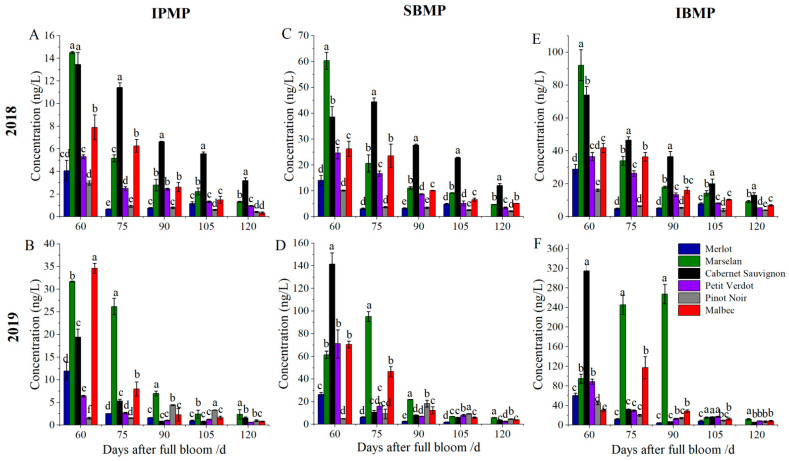
MPs concentrations in grape berries from 6 cultivars during the growth and development. (**A**,**B**) 3-Isopropyl-2-methoxypyrazine (IPMP) concentration in 2018 and 2019; (**C**,**D**) 3-sec-butyl-2-methoxypyrazine (SBMP) concentration in 2018 and 2019; (**E**,**F**) 3-isobutyl-2-methoxypyrazine (IPMP) concentration in 2018 and 2019. Three biological replicates were performed. Bars graphs and error bars represent average and SE, respectively. Significant differences were indicated by lowercase letters based on Fisher LSD-test between different grape varieties of the same sampling period.

The MPs levels of the grape berries from 6 red grape cultivars were determined in two consecutive years (2018 and 2019). In general, the MPs levels in 2019 were higher than that in 2018. In both years, the content of MPs gradually declined from veraison to harvest (Figure 4 and Figure 5). At 60 DAFB, the MOMP, MEMP, and ETMP levels of grape berries were the highest in all cultivars. Moreover, the highest MEMP concentration was observed in Merlot berries in both years. For the ETMP, the highest content was observed in Marselan berries, while the lowest content was in Pinot Noir berries. After full maturity, MOMP content was very low (<0.1 ng/L), and there was almost no detectable MOMP content in Cabernet Sauvignon and Malbec berries in both years. MEMP and ETMP were barely detected in Pinot Noir at harvest. The MEMP and ETMP concentration were the highest in Cabernet Sauvignon berries in 2018, which was higher than in 2019 in all six cultivars at harvest (Figure 4).

Similarly, the IPMP, SBMP, and IBMP also showed a decreasing trend after veraison, and there were detected in all grape cultivars at harvest. At 60 DAFB, the IPMP content in Marselan berries was the highest in 2018, while Malbec berries was the highest in 2019. The highest SBMP and IBMP concentration were observed in Marselan berries at 60 DAFB, followed by Cabernet Sauvignon berries, while an opposite pattern was occurred in 2019 (Figure 5). After full maturity, the higher IPMP content was observed in Marselan, Cabernet Sauvignon, and Merlot berries in two consecutive years. Regarding the SBMP content, Cabernet Sauvignon berries was significantly higher than other cultivars in 2018, while the lowest content was observed in Pinot Noir (2.12 ng/L), the content in Cabernet Sauvignon was 5.67-fold compared with that of Pinot Noir. SBMP content of Marselan berries was the highest in 2019, up to 5.93 g/L. For IBMP, there was the highest content in Cabernet Sauvignon berries (13.05 ng/L) in 2018, followed by Marselan, Merlot, and Malbac berries, while Pinot Noir and Petit Verdot were the lowest. All cultivars except Cabernet Sauvignon berries had a similar trend in 2019. At harvest, all the grape berries from all six cultivars except Cabernet Sauvignon showed higher IBMP content in 2019.

**Figure 6:**
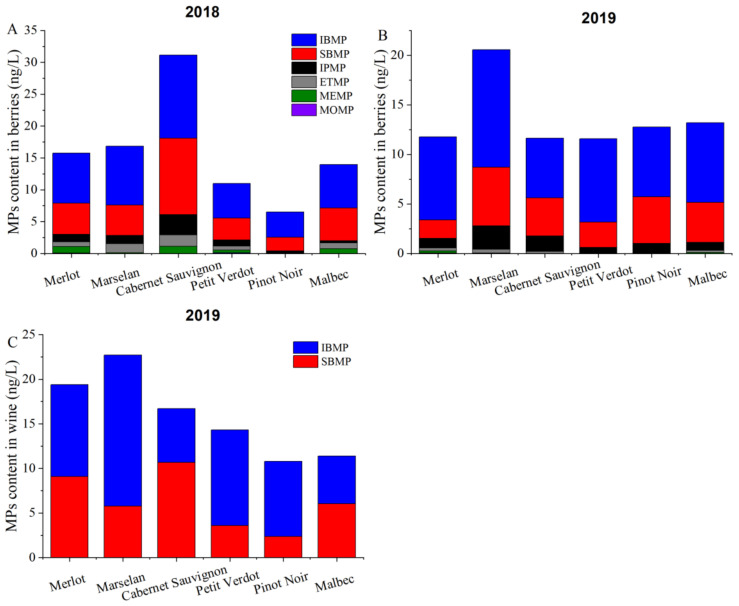
Concentration of total MPs in grape berries at harvest and wine. (**A**) Total MPs content of grape berries at harvest in 2018; (**B**) Total MPs content of grape berries at harvest in 2019; (**C**) Total MPs content of wine in 2019.

Only SBMP and IBMP could be detected in wines, and MPs concentrations were higher in wines than that in the berries. In 2018, at harvest, the highest concentration of total MPs content was observed in Cabernet Sauvignon berries, while Pinot Noir berries had the lowest. In 2019, Marselan berries had the highest content of total MPs, significantly higher than other varieties. For the wines, total MPs content of grape berries in six cultivars in 2019 were different. In short, the MPs level in Cabernet Sauvignon, Marselan, and Merlot berries were higher, while MPs content in Petit Verdot, Malbec, and Pinot Noir berries were relatively lower (Figure 6).

### 2.4. O-Methyltransferase Genes Expression Levels in Grape Berries from Different Cultivars

**Figure 7:**
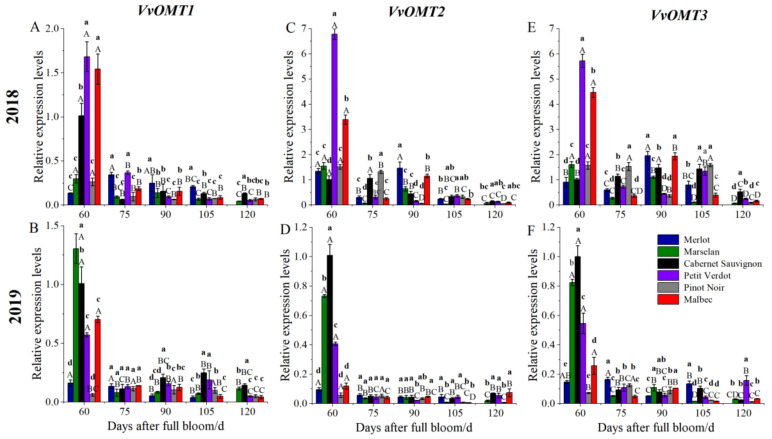
Expression levels of genes related to MPs biosynthesis in grape berries from different cultivars during the growth and development. (**A**,**B**) Expression level of *VvOMT1* in 2018 and 2019; (**C**,**D**) Expression level of *VvOMT2* in 2018 and 2019; (**E**,**F**) Expression level of *VvOMT3* in 2018 and 2019. Three biological replicates were performed. Lines graphs and error bars represent average and SE, respectively. Significant differences were indicated by lowercase letters based on Fisher LSD-test between different grape varieties of the same sampling period. Capital letters were used to indicate significant differences between different sampling periods for the same grape variety (*p* ≤ 0.05).

Differences existed in the content of MPs in different cultivars, while MPs content is determined by its rates of synthesis and degradation. In order to understand the difference in MPs content among different cultivars, the expression levels of genes related to MPs biosynthesis, including *VvOMT1*, *VvOMT2*, and *VvOMT3*, were analyzed in 2018 and 2019, respectively. The results showed that the expression level of *VvOMT1* reached a maximum at 60 DAFB in 2018 and 2019 and then rapidly declined. Furthermore, the expression pattern of *VvOMT2* and *VvOMT3* in grape berries of each cultivar were consistent with those in *VvOMT1*. Regarding the different cultivars, the expression levels of *VvOMT1*, *VvOMT2* and *VvOMT3* in Petit Verdot and Malbec berries were significantly higher than that in Cabernet Sauvignon berries at 60 DAFB in 2018. The expression levels of *VvOMT1* in Marselan was 1.31-fold compared with the Cabernet Sauvignon berries on 60 DAFB in 2019. For *VvOMT2* and *VvOMT3*, Cabernet Sauvignon has the highest expression levels on 60 DAFB. After 60 DAFB, the expression level of *VvOMT3* was significantly higher than that of *VvOMT1* and *VvOMT2* in different cultivars in 2018 (Figure 7).

**Figure 8:**
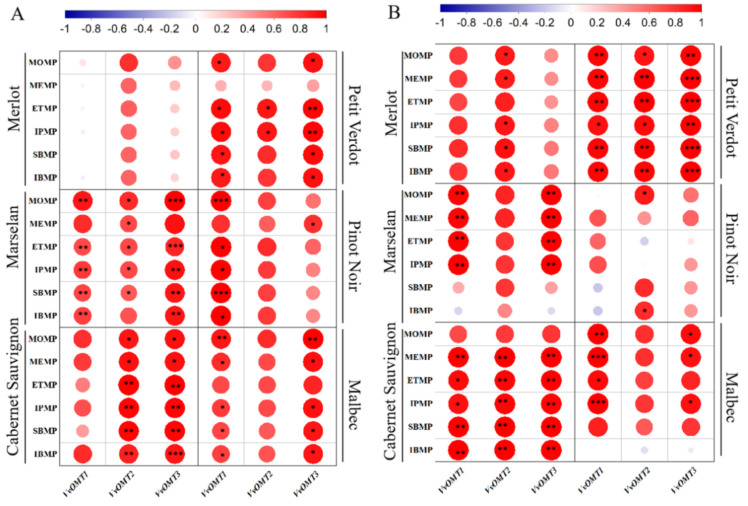
Correlation analysis of MPs concentrations with the expression levels of *VvOMTs* gene in grape berries. (**A**) The correlation analysis of MPs concentrations with the expression levels of *VvOMTs* gene in 2018. (**B**) The correlation analysis of MPs concentrations with the expression levels of *VvOMTs* gene in 2019. Significant correlation were tested by one-way ANOVA and Tukeys post hoc test, “*” indicates significant correlation (*p* ≤ 0.1), “**” indicates significant correlation (*p* ≤ 0.05), “***” represents extremely significant difference (*p* ≤ 0.01).

Furthermore, the correlation between MPs content and expression levels of genes was analyzed. The MPs content of Merlot berries was positively correlated with the expression levels of *VvOMT1*, *VvOMT2*, and *VvOMT3*, among them, the correlation with *VvOMT2* was the highest. A similar relationship was observed in Cabernet Sauvignon and Petit Verdot berries. However, there was a weak correlation in Pinot Noir berries. In general, *VvOMT1* and *VvOMT3* showed the highest correlation with MPs content in grape berries, while *VvOMT2* showed a relatively weak correlation (Figure 8).

## 3. Discussion

The climatic regimes of the experimental site were markedly different in 2018 and 2019. The average daily temperature sum (1067.7 °C) before veraison in 2018 was higher than that in 2019 (1015.5 °C); this may be the reason for the early veraison in 2018. A study of daily temperature and rainfall in Bordeaux on Merlot and Cabernet Sauvignon found that the anthesis of grapevine was earlier with the increase in accumulated temperature, indicating that the increase in sunshine promoted photosynthesis and inflorescence differentiation. However, more rainfall would delay the budding and flowering stage. Obviously, the influence on the early phenological period leads to the occurrence of veraison [24]. The Weight of 100 berries exhibited a gradual upward trend during berry development; this phenomenon has been verified by predecessors [25]. However, there was a decreasing trend at maturity, which may have been due to the control of water to accumulate effective components in the later stage of maturity. At 60 DAFB in 2018, the total soluble solids (TSS) content was higher than that in 2019, and the titratable acid (TA) was lower. This is probably attributed to higher accumulated temperature in 2018, which further resulted in earlier maturity compared with 2019. The rate of TSS accumulation was relatively rapid in Merlot, which may be the main reason for the earlier maturity. A similar phenotype was observed in the Argentina region; it was found that the TA content of Merlot berries was lower than Malbec and Cabernet Sauvignon berries [26]. Anthocyanin function as protectants under biotic and abiotic stress in plants, promoting red-purple coloration of horticultural plant organs, which is also a key parameter in determining external quality. Anthocyanins accumulate continuously in the berries from different cultivars during the growth and development of all cultivars, the regulation was consistent with Villegas [27]. In 2018 and 2019, the total phenol content of Marselan berries was the highest, while an opposite result was observed in Malbec berries. It may be due to the large volume of Malbec berries, which resulted in a lower concentration in unit volume. However, Marselan may be one of the most potential cultivars of wine grapes in China, and Marselan berries composition has not been reported.

In grape berries, MPs exists in six volatile forms and impart a green odor to the grape berries. The content of MPs in wine is closely related to grape berries [10]. In this study, MPs content of Merlot, Cabernet Sauvignon, Marselan, Petit Verdot, Pinot Noir, and Malbec berries from veraison to ripening was determined. The 2-methoxypyrazine (MOMP), 3-methyl-2-methoxypyrazine (MEMP), and 3-ethyl-2-methoxypyrazine (ETMP) levels in grape berries were relatively lower at harvest, and the highest content was only 1.79 ng/L (ETMP) of Cabernet Sauvignon in 2019. While the 3-isopropyl-2-methoxypyrazine (IPMP), 3-sec-butyl-2-methoxypyrazine (SBMP), and 3-isobutyl-2-methoxypyrazine (IBMP) levels in berries were higher, we found that most of the research only focused on IBMP in Cabernet Sauvignon [12,16,28]. The odor spectra of alkyl pyrazine and 3-substituted 2-alkyl pyrazine were determined by Masuda; generally, the odor threshold of alkyl pyrazines decreases with the increase in the number of side-chain carbon atoms; the sensory thresholds of MOMP, MEMP, and ETMP have been reported as 400,000 ng/L, 7000 ng/L, and 10,000 ng/L, respectively [29]. In this study, the results indicated that the MOMP, MEMP, and ETMP content of berries was below the sensory threshold, indicating no effect on the flavor of berry and wine. Meanwhile, IPMP, SBMP, and IBMP are the most abundant MPs in fruits. IPMP is the main volatile that imparts asparagus and pea [1] and the odor of roots, soils, and potatoes [30]. In this study, the highest IPMP content in Cabernet Sauvignon and Marselan berries was found at harvest, but no IPMP was detected in wines. SBMP is mainly found in vegetables, such as sweet root and carrot [31]. SBMP content was undetectable in Cabernet Sauvignon wines, and therefore there was no adverse effect in other literatures [1,32]. However, the SBMP content was higher at harvest in the present study. IPMP, SBMP, and IBMP in distilled water at 1–2 ng/L concentration can be detected by the nose [28]. Among the six MPs, the content of IBMP was the highest at harvest, with values exceeding the threshold and having the most significant impact on grapes, this observation is consistent with earlier reports [33,34]. Thus, it can be concluded that IPMP, SBMP, and IBMP contribute to the foul smell of grape berries; meanwhile, SBMP and IBMP may have a negative impact on the quality of various varieties of wine.

In previous studies, MPs have been identified in Cabernet Sauvignon, Merlot, Malbec, and Pinot Noir berries, of which Cabernet Sauvignon berries was the most widely studied [12,16,28]. In this study, higher IBMP content was observed in Cabernet Sauvignon, Malbec, Merlot, and Marselan berries. In Malbec must, the IBMP content was lower than the lower limit of quantification [11], which may be due to the lower IBMP content in pulp, while higher IBMP content was observed in seeded fruit. The strong green pepper odor in Cabernet Sauvignon, Cabernet Franc, and Merlot berries occurred due to variety specificity. Additionally, Cabernet Sauvignon berries with an obvious robust plant characteristic aroma resulted from IBMP [19], which is consistent with the study in Cabernet Sauvignon in 2018. However, IBMP was slightly lower in Merlot than Cabernet Sauvignon in Japan [17]. The content of IBMP in Pinot Noir red wines from France and Australia was lower [35], consistent with the current study. However, the MPs of Marselan berries has not been reported. To evaluate the correlation between the decrease in IPMP and IBMP levels and the dilution effect caused by the increase in berry volume of different cultivars, the absolute content of each compound was calculated (pg/berry; the average fresh weight of berry) [5,8]. However, in this study, the single berry weight showed a stable trend after veraison. Therefore, the concentration per unit volume may also reflect the increase or decrease in MPs in a single berry.

Furthermore, the study found that MPs in grape berries were mainly accumulated before veraison (22 July), then degraded rapidly and reached a low level at harvest, which is similar to the reports by Roujou de boubée [16]. During the development, a slight increase in MPs was observed for a short period, probably because the analysis of internal standards was unstable; therefore, an appropriate internal standard is required. Another possible reason for this slight increase may be the mutual transformation of MPs and hydroxy pyrazine [36,37]. The decline in MPs content of Merlot berries during ripening is slowly, probably due to Merlot fruit had turned color and passed the rapid decline period.

The IBMP and SBMP levels in Cabernet Sauvignon and Marselan berries were higher and showed a downward trend during development. However, in 2019, IBMP and SBMP of Marselan berries showed a trend of first rising and then declining; the highest value appeared in August, indicating that Marselan is a late maturing cultivar. Furthermore, MOMP, MEMP, ETMP, and IPMP were not detected in the wine of any grape cultivars; the SBMP and IBMP content was 2–16 ng/L, consistent with Sidhu [13]. The sensory threshold in wine is usually 2 ng/L [19]. The SBMP and IBMP contents of berries at harvest were lower than those in wine, probably because MPs in the seeds or other tissues dissociated during soaking [28]. Additionally, the high temperature and alcohol under fermentation made it easier for MPs to volatilize into wine, thus increasing the content of MPs in the wine [31]. The IBMP content of different wine grape cultivars at harvest was similar to that in wine, consistent with the result from Harris [38]. The content of IBMP in all wines was higher than the threshold value. In order to reduce IBMP content in berries, various cultivation measures such as changing the training system, leaf picking, reducing irrigation, and reducing yield have been adopted. Removing fruit stalks and reducing maceration time during brewing are other potential methods to reduce IBMP in wine [6,8,12,28,31,39].

To date, researchers have identified four *OMT* genes (*VvOMT1*, *VvOMT2*, *VvOMT3* and *VvOMT4*) involved in MP biosynthesis [23,40,41]. In this study, the transcription levels of *VvOMT1*, *VvOMT2*, and *VvOMT3* decreased rapidly after veraison, consistent with earlier reports [5,8,40]. Results from correlation analysis demonstrated that the strongest association between MPs and the expression levels of *VvOMT1* and *VvOMT3* were in Marselan, Cabernet Sauvignon, and Malbec berries. A relatively weaker correlation was observed in *VvOMT2*; on the one hand, it may be due to spatial and temporal difference between the change in MPs content and genes expression, on the other hand, the highest expression level of *VvOMT2* was observed in the root [42]. Meanwhile, MPs content in Merlot and Petit Verdot berries strongly correlated with *VvOMT1* and *VvOMT2*, respectively. *VvOMT1*, *VvOMT2*, and *VvOMT3* had a significant effect on the accumulation of IPMP and a poor correlation with IBMP. Gregan also found that the highest expression of these three genes was closely related to the time when the IPMP levels in berries was the highest in the past two years [5]. Although we understand the relationship between the difference in MPs components, level and gene expression in different varieties at the gene level, we still need to further explore the tissue specificity of *VvOMTs* genes expression.

## 4. Materials and Methods

### 4.1. Experimental Site and Grape Materials

The experiment was carried out in the commercial vineyards of the Jinshan area in the eastern foothill of the Helan Mountain (37°43′–39°23′ N, 105°45′–106°47′ E) during the 2018–2019 growing season. The area has a typical continental climate and lime calcareous and sandy loam soils. Merlot, Cabernet Sauvignon, Marselan, Petit Verdot, Pinot Noir, and Malbec wine grape cultivars were used as experimental materials. All the grapevines were self-rooted seedlings, the row spacing is 3.0 m, and the in-raw distance between plants is 1.0 m. The vineyard management measures were in accordance with local agronomic practices. The meteorological monitoring station was installed in Meihe Manor (38°37′ N, 106°01′–106°47′ E) from the vineyard. Effective accumulated temperature = Σ(Daily mean temperature − Biological lower limit temperature), the biological lower limit temperature was 10 °C in the vine.

A total of 60 grapevines with vigorous growth conditions and similar size were selected for each biological replicate and three biological replicates were performed in this study. The full bloom period of Cabernet Sauvignon is 25 May in 2018 and 29 May in 2019. The berries samples were collected at 60, 75, 90, 105 and 120 DAFB in 2018 and 2019. All samplings were harvested at 120 DAFB, unless Merlot was harvest at 110 DAFB due to its early maturity. For each cultivar, 300 grape berries from 10 different plants were collected, and mixed into a single sample. The weight of 100 berries were determined firstly, then 100 grape berries were squeezed into juice and the supernatant was used to determine the total soluble solid and titratable acid content. Other berries samples were used for the determination of physical and chemical indices and were immediately frozen in liquid nitrogen and stored in refrigerator at −80 °C.

### 4.2. Winemaking Processes

A total of 10 kg grape berries from each cultivar were collected on 20 September 2019; grapes were de-stemmed, then crushed, and the resulting must was transferred to stainless vessels for fermentation. The fermentation temperature was maintained at 25–28 °C. During fermentation, the wine was stirred and the specific gravity of grape must was determined every day until it became constant. Skins and seeds were separated after fermentation. At the end of alcoholic fermentation, malolactic fermentation was carried out.

### 4.3. Determination of Berry Quality

The weight of 100 berries was measured using the one ten-thousandth balance (ME 203E, Mettler Toledo, Zurich, Switzerland). The berries were squeezed into juice and a supernatant was used to determine the total soluble solid (TSS) and titratable acid (TA) content. TSS was determined using a WYT 24 hand-held refractometer (Zhengjin Instrument Equipment, HuBei, China). The sodium hydroxide titration (0.05 M) was used to determine the titratable acid. The anthocyanin content was quantified through the differential pH method [43]. The total phenols in the berries were extracted with Folin–Ciocalteu (F-C) [44], and the berry tannin content was determined using Folin–Denis (F-D) [45].

### 4.4. Solution and Sample Preparation for MP Determination

The simulated grape juice was prepared by mixing glucose and fructose at a 1:1 ratio in deionized water to a final concentration of 200 g/L; the pH was adjusted to 3.5 with tartaric acid. The MP standards, including MOMP, MEMP, ETMP, IPMP, SBMP and IBMP were diluted with anhydrous ethanol to prepare a single standard solution of 200 mg/L concentration. Added to the simulated grape juice in single standard solution at the final concentration of 2.0 mg/L working solution of each standard was prepared and stored in the refrigerator at 4 °C in the dark.

Approximately 30 g of berries stored at −80 °C were weighed and put into the crushing cup of a tissue crusher and mixed with 0.3 g of PVPP. After crushing, the mixture was allowed to melt at 4 °C and centrifuged at 5000 rpm for 10 min. Then, 5 mL of the collected grape juice was added into a 15-mL brown headspace bottle with 5 μL of 2-acetylpyridine (internal standard, concentration 500.0 μg/L) and 2.0 g NaCl, and closed with the bottle cap. For wine, 5 mL of wine sample was added into a 15-mL brown headspace bottle, as described above.

### 4.5. Gas Chromatography-Mass Spectrometry (GC-MS) Determination of MPs in Grape Berries

The MPs were determined using a GC-MS (Agilent 7890B-5977B, Agilent, California, USA). Chromatographically pure MOMP (CAS: 3149-28-8), MEMP (CAS: 2847-30-5), ETMP (CAS: 25680-58-4), IPMP (CAS: 25773-40-4), SBMP (CAS: 24168-70-5), and IBMP (CAS: 24683-00-9) standards were supplied from the National reference materials network (Beijing, China). The headspace bottle with the sample was placed in the headspace solid phase microextraction device (Shanshan Machinery Company, Tianjin, China), preheated at 40 °C for 5 min, and extracted with 50 μm/30 μm CAR/PDMS extraction head (Sigma Aldrich, ShangHai, China) for 3 h at 40 °C in the dark. The extraction head was inserted into the injection port for desorption for 5 min and detected by GC-MS.

An A J & W DB wax chromatographic column (30 m × 0.25 mm × 0.25 μm; Agilent, California, USA) was used. The injection port temperature was set at 230 °C, and the detector temperature at 250 °C. Helium was used as the carrier gas at a flow rate of 2.0 mL/min, with no split injection. The temperature rising procedure was as follows: an initial hold at 40 °C for 1 min, followed by an increase to 100 °C at the rate of 3 °C/min and hold for 2 min, and then a rise to 230 °C at the rate of 50 °C/min and hold for 10 min. Selected ion monitoring (SIM) mode was used for testing. For qualitative analysis, each MP standard was injected into the headspace bottle with 2 mg/L. Each MP was identified based on the peak time and by comparing the spectra with the NIST library. The internal-standard method was used for relative quantification.

### 4.6. Determination of VvOMTs Gene Expression

Total RNA was obtained using the RNAprep Pure Plant Kit (Polysaccharides and Polyphenolics-rich) (TIANGEN, Beijing, China) according to the manufacturer’s instructions. Purity and integrity of total RNA were detected by 1% agarose electrophoresis and Nanodrop 2000 (NanoDrop Technologies, Wilmington, NC, USA). In total, 1 μg RNA was reverse-transcribed using EasyScript@One-step gDNA Removal and cDNA Synthesis SuperMix (TransGen Biotech, Beijing, China) to obtain the first-strand cDNA.

Analysis of *VvOMT1*, *VvOMT2*, and *VvOMT3* mRNA expression by Quantitative Real-Time PCR (qRT-PCR). The primers used for qRT-PCR were designed using Primer 5.0. The forward and reverse primers are as follows: forward 5′-GAGAAGCGAGGTGGAATG-3′ and reverse 5′-TGAGATGATTACTCTGGATATGC-3′ for *VvOMT1*; forward 5′-CCGAGGTTGAATGGAAGAA-3′ and reverse 5′-AATGACAAACACGATGTAGATTAC-3′ for *VvOMT2*; forward 5′-ATGATGGCTCATACTACTAC-3′ and reverse 5′- CCTAATTTCGTGTCCTAATG-3′ for *VvOMT3*; and forward 5′-CTTGCATCCCTCAGCACCTT-3′, reverse 5′-TCCTGTGGACAATGGATGGA-3 for *VvActin*. *VvActin* (XM_002282480.4) was selected as the internal reference for grape. The relative expression of the genes was analyzed on a real-time PCR system (qTOWER3G, Analylik Jena AG, Germany). The thermal cycling conditions included an initial denaturation at 94 °C for 30 s, followed by 40 cycles of 94 °C for 5 s, 56 °C for 15 s, and 72 °C for 10 s. Three biological replicates were performed. Taking the expression of *VvOMTs* in Cabernet Sauvignon berries for 60 DAFB as a unit, the relative expression of genes in berries of different cultivars was calculated using the 2^−^^ΔΔCt^ method [46].

### 4.7. Statistical Analysis

The data were analyzed using IBM SPSS Statistics 26 (IBM, NY, USA) software. One-way analysis of variance (ANOVA) was used to determine statistically significant differences (*p* ≤ 0.05). Line graphs and bar graphs were plotted in Origin 9.0 (OriginLab Corporation, Northampton, MA, USA) software. Meanwhile, Origin 9.8.5 (OriginLab Corporation, Northampton, MA, USA) software was used to determine the Pearson correlation coefficients, considered significant at 0.1, 0.05 and 0.01.

## 5. Conclusions

The present study evaluated the quality features of 6 kinds of red wine grapes in the Jinshan region of the eastern foothill of Helan Mountain. At harvest, Malbec had the largest berries weight; Merlot and Petit Verdot had high TSS content with low TA content. The highest anthocyanin content was observed in Marselan. Meanwhile, the IBMP level in all cultivars of wines was higher than the sensory threshold, especially in Cabernet Sauvignon, Marselan, Merlot, and Malbec. Therefore, improved cultivation measures or brewing procedures should be adopted to reduce MPs content effectively in these cultivars. However, these improvement measures need further investigation.

## Data Availability

Not applicable.
